# Supplementary material for: Mechanical Constraint Effect on DNA Persistence Length
Source: Molecules. 2022 Nov 11;27(22):7769. doi: 10.3390/molecules27227769 (PMC9696218; doi:10.3390/molecules27227769)
Supplement: Supplementary file 1 [file molecules-27-07769-s001.zip › molecules-1940123-supplementary.pdf]

Supplementary Information

File SI: Derivation of critical buckling length of DNA fragment with elastic end-constraints

A selected DNA fragment with flexural rigidity *EI*, length l_{cb} with elastic end-constraints subjected to an axial compressive load *P* as shown in Figure S1. Here the mechanical constraints for the selected DNA fragment provided by other fragments are simplified as rotational springs and lateral springs, with spring constants $\overline{k}_{rsA}$ and $\overline{k}_{lsA}$ for rotational spring and lateral spring at end $\overline{x}=0$, and $\overline{k}_{rsB}$ and $\overline{k}_{lsB}$ for rotational spring and lateral spring at end $\overline{x}=l_{cb}$, respectively. $\overline{x}$ is the axial coordinate of DNA fragment.

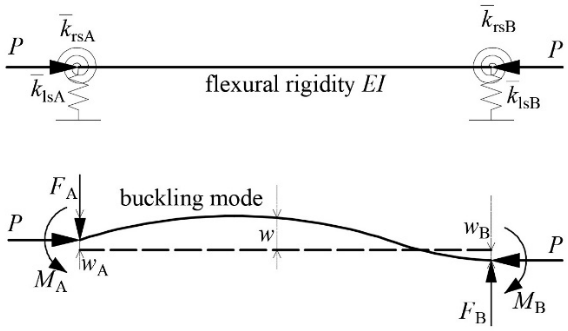

Figure S1 Buckling of compressed DNA fragment with elastic end-constraints

According to the second order differentiation of deflection and equilibrium equation of the compressed fragment, a normalized governing equation for the buckled deformation of Euler beam can be expressed as [1]

$$\frac{d^4 w}{dx^4}+\alpha^2\frac{d^2 w}{dx^2}=0,\quad \alpha^2=\frac{Pl_{cb}^2}{EI}, \tag{S1}$$

where $w=\overline{w}/l_{cb}$ and $x=\overline{x}/l_{cb}$, and $\overline{w}$ is the transverse deflection at $\overline{x}$.

The general solution for Eq. (S1) is given as

$$w=C_1\sin\alpha x+C_2\cos\alpha x+C_3 x+C_4, \tag{S2}$$

in which the four constants C_i (i=1,2,3,4) can be evaluated by the following boundary conditions

at each end of DNA fragment, x=0 and x=1.

The boundary conditions at x=0 are given as

$$\frac{\bar{k}_{\text{rsA}}}{EI}\left(\frac{\mathrm{d}w}{\mathrm{d}x}\right)_{x=0}-\left(\frac{\mathrm{d}^2 w}{\mathrm{d}x^2}\right)_{x=0}=0,\ \frac{\bar{k}_{\text{lsA}}}{EI}\left(w\right)_{x=0}-\left(\frac{\mathrm{d}^3 w}{\mathrm{d}x^3}\right)_{x=0}=0, \tag{S3}$$

and those at x=1 are given as

$$\frac{\bar{k}_{\text{rsB}}}{EI}\left(\frac{\mathrm{d}w}{\mathrm{d}x}\right)_{x=1}+\left(\frac{\mathrm{d}^2 w}{\mathrm{d}x^2}\right)_{x=1}=0,\ \frac{\bar{k}_{\text{lsB}}}{EI}\left(w\right)_{x=0}+\left(\frac{\mathrm{d}^3 w}{\mathrm{d}x^3}\right)_{x=1}=0. \tag{S4}$$

It is set $\frac{\bar{k}_{\text{rsA}}}{EI}=k_{\text{rsA}}$ and so on for convenience. By substituting Eq. (S2) into boundary conditions Eqs. (S3) and (S4), then doing simplification, a matrix equation is expressed as

$$\begin{bmatrix} \alpha k_{\text{rsA}} & \alpha^2 & k_{\text{rsA}} & 0 \\ \alpha^3 & k_{\text{lsA}} & 0 & k_{\text{lsA}} \\ \left(\alpha^2\sin\alpha+\alpha k_{\text{rsB}}\cos\alpha\right) & \left(\alpha^2\cos\alpha-\alpha k_{\text{rsB}}\sin\alpha\right) & k_{\text{rsB}} & 0 \\ \left(\alpha^3\cos\alpha+k_{\text{lsB}}\sin\alpha\right) & \left(-\alpha^3\sin\alpha+k_{\text{lsB}}\cos\alpha\right) & k_{\text{lsB}} & k_{\text{lsB}} \end{bmatrix}\begin{bmatrix} C_1 \\ C_2 \\ C_3 \\ C_4 \end{bmatrix}=\begin{bmatrix} 0 \\ 0 \\ 0 \\ 0 \end{bmatrix}. \tag{S5}$$

The necessary and sufficient condition for non-zero solution of constants C_i for the above homogeneous linear equations Eq. (S5) is that its determinant in matrix is equal to zero, i.e.

$$\begin{vmatrix} \alpha k_{\text{rsA}} & \alpha^2 & k_{\text{rsA}} & 0 \\ \alpha^3 & k_{\text{lsA}} & 0 & k_{\text{lsA}} \\ \left(\alpha^2\sin\alpha+\alpha k_{\text{rsB}}\cos\alpha\right) & \left(\alpha^2\cos\alpha-\alpha k_{\text{rsB}}\sin\alpha\right) & k_{\text{rsB}} & 0 \\ \left(\alpha^3\cos\alpha+k_{\text{lsB}}\sin\alpha\right) & \left(-\alpha^3\sin\alpha+k_{\text{lsB}}\cos\alpha\right) & k_{\text{lsB}} & k_{\text{lsB}} \end{vmatrix}=0, \tag{S7}$$

or in a form of a transcendental equation expressed as

$$\begin{aligned} &-\alpha\left[k_{\text{lsB}}k_{\text{rsB}}\alpha^4+k_{\text{lsA}}k_{\text{rsA}}\left(\alpha^4-2k_{\text{lsB}}k_{\text{rsB}}\right)\right]- \\ &\alpha\left[2k_{\text{lsA}}k_{\text{rsA}}k_{\text{lsB}}k_{\text{rsB}}+k_{\text{lsA}}k_{\text{lsB}}\left(k_{\text{rsB}}-k_{\text{rsA}}\right)\alpha^2-\left(k_{\text{lsB}}k_{\text{rsA}}+k_{\text{lsA}}k_{\text{rsB}}\right)\alpha^4\right]\cos\alpha- \\ &\alpha^2\left[k_{\text{lsA}}k_{\text{lsB}}\left(k_{\text{rsA}}+\left(k_{\text{rsA}}-1\right)k_{\text{rsB}}\right)+\left(k_{\text{lsA}}k_{\text{lsB}}+\left(k_{\text{lsB}}-k_{\text{lsA}}\right)k_{\text{rsA}}k_{\text{rsB}}\right)\alpha^2\right]\sin\alpha=0. \end{aligned} \tag{S8}$$

If the spring constant $\bar{k}_{\text{rsA}}$, $\bar{k}_{\text{lsA}}$, $\bar{k}_{\text{rsB}}$, $\bar{k}_{\text{lsB}}$ and fragment flexural rigidity EI are given, the numerical solution of the smallest α in Eq. (S8) will be obtained, in other words, α is dependent on the elastic end-constraints of fragment.

After determining α, noting the above definition $\alpha^2=\frac{Pl_{\text{cb}}^2}{EI}$ in Eq. (S2), the critical buckling

length of fragment is expressed as

$$l_{\text{cb}} = \alpha\sqrt{\frac{EI}{P}}\,. \tag{S9}$$

When the spring constants of both lateral springs, k_{lsA} and k_{lsB}, are infinity, and spring constants of both rotational springs, k_{rsA} and k_{rsB}, are zero, this elastic end-constraint degenerate to a classical specific end-constraint, hinge-hinge end-constraint, and Eq. (S8) also degenerate to

$$\alpha^4 \sin\alpha = 0\,, \tag{S10}$$

α=0 is a meaningless solution of Eq. (S10) for buckling problem, so $\alpha=\pi$ is the minimum meaningful solution of Eq. (S10). Therefore, the critical buckling length for hinge-hinge end-constraint is

$$l_{\text{cb-hh}} = \pi\sqrt{\frac{EI}{P}}\,. \tag{S11}$$

Eq. (S11) was first derived by Leonhard Euler and is called as Euler's formula. The ratio of the critical buckling length with hinge-hinge end-constraint to that with any elastic end-constraint is defined as effective length factor μ expressed as

$$\mu = \frac{l_{\text{cb-hh}}}{l_{\text{cb}}} = \frac{\pi}{\alpha}\,. \tag{S12}$$

To be formally consistent with the Euler's formula, Eq. (S9) is rewritten with effective length factor μ as

$$l_{\text{cb}} = \frac{\pi}{\mu}\sqrt{\frac{EI}{P}}\,. \tag{S13}$$

File SII: Comparisons of fitting curves and experimental results

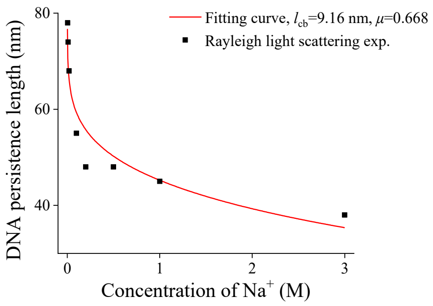

Figure S2 Fitting curve and Rayleigh light scattering experimental result [2] for 39000 bp DNA in NaH_2PO_4 solution, goodness of fit = 0.893

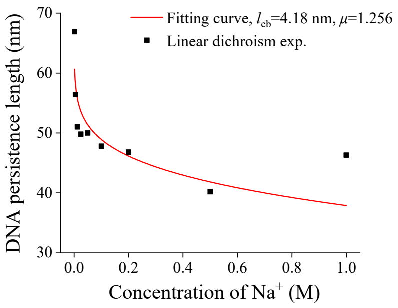

Figure S3 Fitting curve and linear dichroism experimental result [3] for 39000 bp DNA in NaCl solution, goodness of fit = 0.652

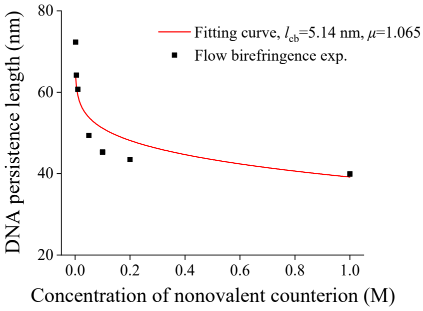

Figure S4 Fitting curve and flow birefringence experimental result [4] for 39000 bp DNA in NaCl solution, goodness of fit = 0.815

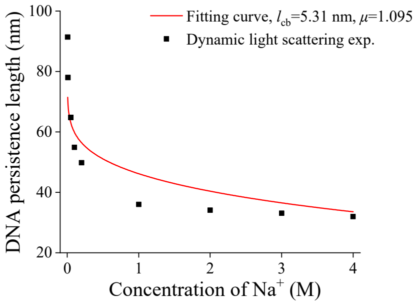

Figure S5 Fitting curve and dynamic light scattering experimental result [5] for 6594 bp DNA in NaCl solution, goodness of fit = 0.815

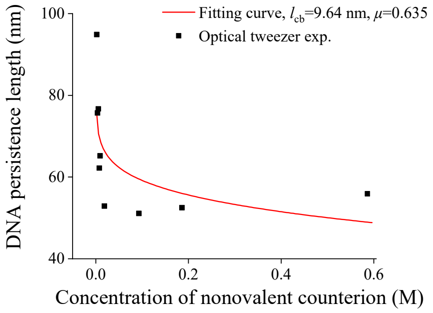

Figure S6 Fitting curve and optical tweezer experimental result [6] for 48500 bp DNA in NaCl solution, goodness of fit = 0.511

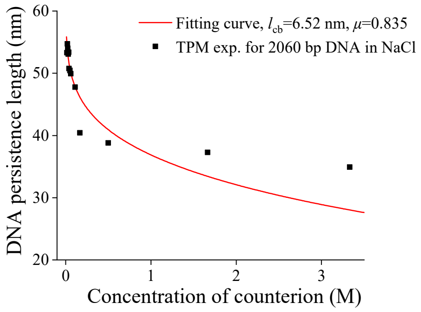

Figure S7 Fitting curve and TPM experimental result [7] for 2060 bp DNA in NaCl solution, goodness of fit = 0.824

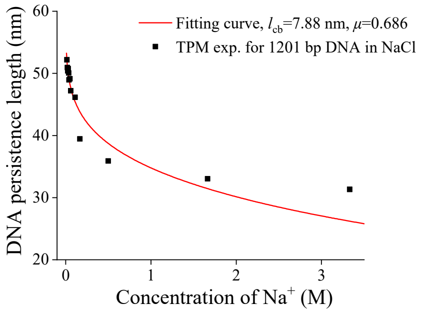

Figure S8 Fitting curve and TPM experimental result for [7] 1201 bp DNA in NaCl solution, goodness of fit = 0.911

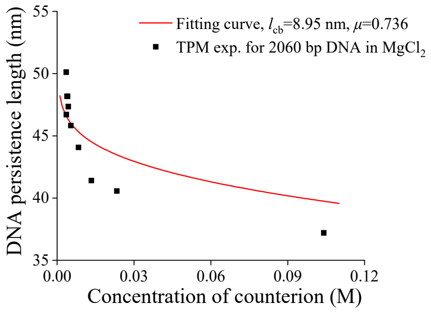

Figure S9 Fitting curve and TPM experimental result [7] for 2060 bp DNA in $MgCl_2$ solution, goodness of fit = 0.614

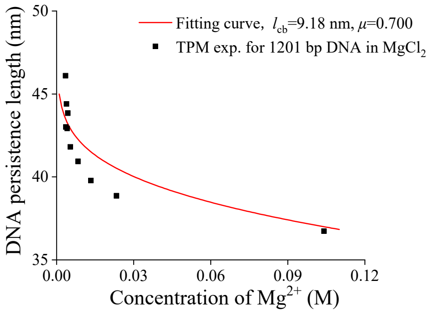

Figure S10 Fitting curve and TPM experimental result [7] for 1201 bp DNA in $MgCl_2$ solution, goodness of fit = 0.624

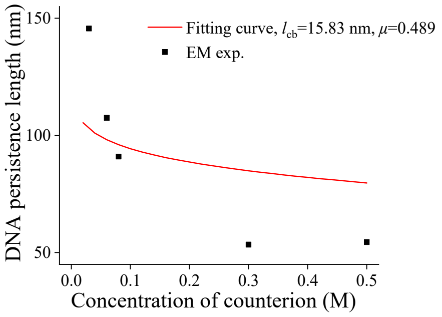

Figure S11 Fitting curve and EM experimental result [8] for 4800 bp DNA in NaCl solution, goodness of fit = 0.404

File SIII: DNA conformation and persistence length versus MD simulation time

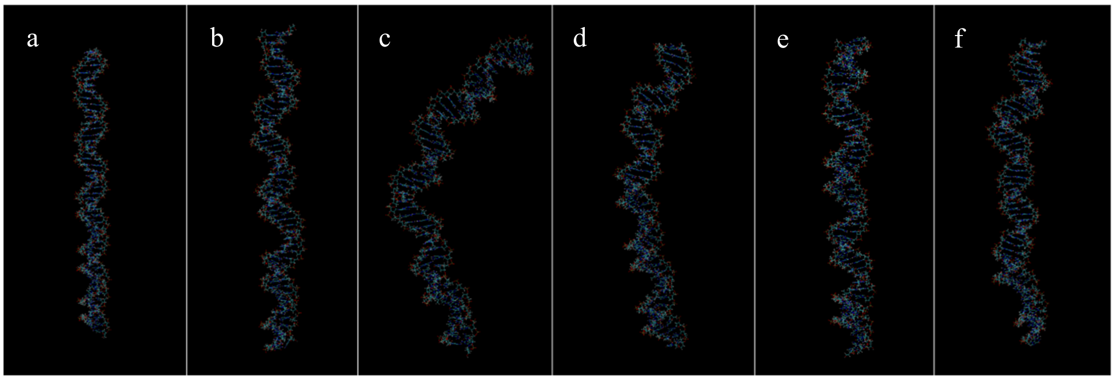

Figure S12 DNA conformations by MD simulation at (a) 0 ns, (b) 10 ns, (c) 20 ns, (d) 30 ns, (e) 40 ns and (f) 50 ns with 0.1 M Na^+, 298K condition

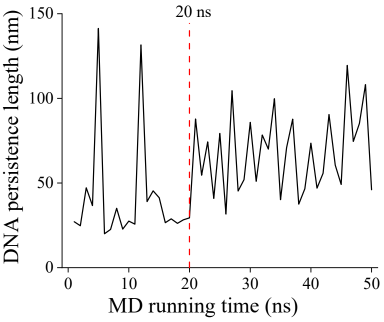

Figure S13 DNA persistence length by MD versus MD running time with 0.01 M Na^+, 298K condition

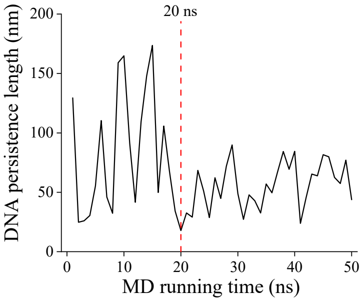

Figure S14 DNA persistence length by MD versus MD running time with 0.1 M Na^+, 298K condition

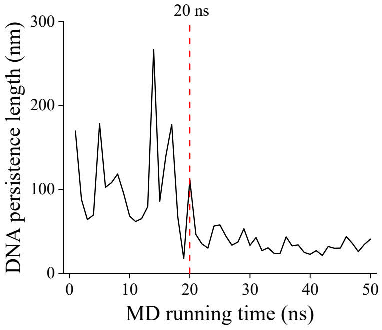

Figure S15 DNA persistence length by MD versus MD running time with 1.0 M Na^{+}, 298K condition

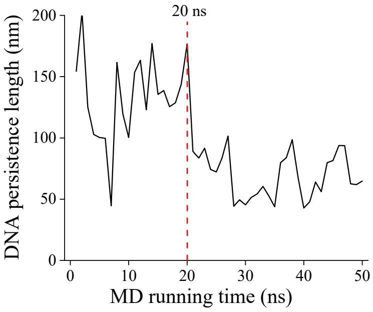

Figure S16 DNA persistence length by MD versus MD running time with 0.1 M Na^{+}, 278K condition

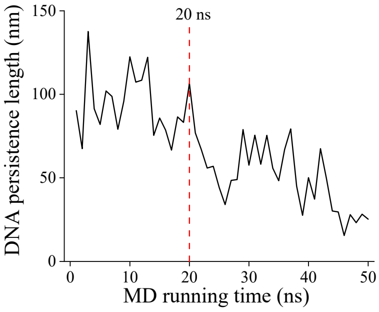

Figure S17 DNA persistence length by MD versus MD running time with 0.1 M Na^{+}, 315K condition

References

1. Bažant, Z. P.; Cedolin, L., *Stability of Structures: Elastic, Inelastic, Fracture and Damage Theories*. World Scientific Publishing Co. Pte. Ltd.: Singapore, 2010.
2. Sobel, E.; Harpst, J., Effects of Na^{+} on the persistence length and excluded volume of T7 bacteriophage DNA. *Biopolymers* **1991,** 31, (13), 1559–1564.
3. Rizzo, V.; Schellman, J., Flow dichroism of T7 DNA as a function of salt concentration. *Biopolymers* **1981,** 20, (10), 2143–2163.
4. Cairney, K. L.; Harrington, R. E., Flow birefringence of T7 phage DNA: dependence on salt concentration. *Biopolymers* **1982,** 21, (5), 923–34.
5. Borochov, N.; Eisenberg, H.; Kam, Z., Dependence of DNA conformation on the concentration of salt. *Biopolymers* **1981,** 20, (1), 231–235.

6. Baumann, C. G.; Smith, S. B.; Bloomfield, V. A.; Bustamante, C., Ionic effects on the elasticity of single DNA molecules. *Proc. Natl. Acad. Sci. U. S. A.* **1997,** 94, (12), 6185–6190.
7. Brunet, A.; Tardin, C.; Salomé, L.; Rousseau, P.; Destainville, N.; Manghi, M., Dependence of DNA persistence length on ionic strength of solutions with monovalent and divalent salts: a joint theory–experiment study. *Macromolecules* **2015,** 48, (11), 3641–3652.
8. Frontali, C.; Dore, E.; Ferrauto, A.; Gratton, E.; Bettini, A.; Pozzan, M. R.; Valdevit, E., An absolute method for the determination of the persistence length of native DNA from electron micrographs. *Biopolymers* **1979,** 18, (6), 1353–73.
